# In Vivo Corneal Microstructural Changes in Herpetic Stromal Keratitis: A Spectral Domain Optical Coherence Tomography Analysis

**DOI:** 10.18502/jovr.v15i3.7446

**Published:** 2020-07-29

**Authors:** Alejandro Rodriguez-Garcia, Raul Alfaro-Rangel, Andres Bustamante-Arias, Julio C. Hernandez-Camarena

**Affiliations:** ^1^Tecnologico de Monterrey, School of Medicine and Health Sciences, Institute of Ophthalmology and Visual Sciences, Cornea and External Diseases Service, Monterrey, Mexico

**Keywords:** Corneal Infection, Herpetic Keratitis, HSV-1, SD-OCT, Stromal Edema

## Abstract

**Purpose:**

To describe and analyze the microstructural changes in herpetic stromal keratitis (HSK) observed *in vivo* by spectral-domain ocular coherence tomography (SD-OCT) at different stages of the disease.

**Methods:**

A prospective, cross-sectional, observational, and comparative SD-OCT analysis of corneas with active and inactive keratitis was performed, and the pathologic differences between the necrotizing and non-necrotizing forms of the disease were analyzed.

**Results:**

Fifty-three corneas belonging to 43 (81.1%) women and 10 (18.8%) men with a mean age of 41.0 years were included for analysis. Twenty-four (45.3%) eyes had active keratitis, and 29 (54.7%) had inactive keratitis; the majority (83.0%) had the non-necrotizing form. Most corneas (79.1%) with active keratitis showed stromal edema and inflammatory infiltrates. Almost half of the active lesions affected the visual axis, were found at mid-stromal depth, and had a medium density. By contrast, corneas with inactive keratitis were characterized by stromal scarring (89.6%), epithelial remodeling (72.4%), and stromal thinning (68.9%). In contrast to non-necrotizing corneas, those with necrotizing HSK showed severe stromal scarring, inflammatory infiltration, and thinning. Additionally, most necrotizing lesions (77.7%) affected the visual axis and had a higher density (*P* = 0.01).

**Conclusion:**

Active HSK is characterized by significant epithelial and stromal thickening and the inactive disease manifests epithelial remodeling at sites of stromal thinning due to scarring. Necrotizing keratitis is characterized by distorted corneal architecture, substantial stromal inflammatory infiltration, and thinning. *In vivo* SD-OCT analysis permitted a better understanding of the inflammatory and repair mechanisms occurring in this blinding corneal disease.

##  INTRODUCTION

Herpetic keratitis is the major cause of corneal blindness in many developed countries, with the stromal form representing almost one-third (29.5%) of all cases.^[1,2]^ Herpetic stromal keratitis (HSK) accounts for up to 44% of recurrences, and the risk for recurrent infection increases after multiple attacks of keratitis.^[3,4]^ Therefore, HSK represents a significant burden of ocular disease, being the most feared presentation of herpetic corneal infection due to its severe damage to the cornea.^[5]^ Most cases of HSK present as non-necrotizing.^[6]^ Non-necrotizing HSK or immune stromal keratitis is characterized by stromal inflammation leading to scarring, thinning, and vascularization of the cornea.^[7,8]^ The direct viral antigen stimulation of HSV-1-specific CD4αβ T-lymphocytes probably drives stromal inflammation. Other likely pathogenic mechanisms are autoantigens unmasked and mimicked by HSV-1 corneal infection, bystander cytokine activation of CD4αβ T-cells, or a combination of all these mechanisms.^[9,10,11]^ On the other hand, the necrotizing form is characterized by ulcerations, dense leukocytic stromal infiltration, and necrosis that may rapidly progress to corneal perforation.^[9,10]^ Both viral antigens and replicating virions have been implicated in the pathogenesis of this form of keratitis.^[12,13]^

Most of our knowledge of the pathologic alterations seen in HSK is based on direct slit-lamp observations and histopathologic findings from fixed tissues.^[14,15]^ Recently, *in vivo* confocal microscopy analysis of herpetic keratitis has shown that a significant and gradual decrease in superficial epithelial cell density is correlated with decreased corneal innervation.^[16]^ The same imaging technique has also been used to monitor the inflammatory process of HSK.^[17]^ Spectral-domain optical coherence tomography (SD-OCT) provides noncontact *in vivo* corneal cross-sectional, high-resolution images that allow a detailed delineation of the cornea.^[18]^ The high speed of the technique permits the acquisition of high-definition frames with few motion artifacts.^[19,20]^ Enlarged sections of averaged images permit the discrimination of all corneal layers.^[21]^ SD-OCT also enables more accurate measurements of the corneal thickness at a particular point, or in a 6 mm diameter pachymetry map even in the presence of corneal irregularity or opacity.^[8,22]^

The primary purpose of the present study was to describe and analyze the microstructural corneal alterations observed by SD-OCT during the active and inactive stages of HSK and to compare those changes between non-necrotizing and necrotizing keratitis. This analysis adds to our understanding of the inflammatory and repair mechanisms of the cornea in this sight-threatening disease.

##  METHODS

This is a prospective, cross-sectional, descriptive, comparative, and observational study of patients diagnosed with HSK based on clinical findings. The inclusion criteria for the disease diagnosis were based on previous medical history, clinical manifestations, and therapeutic response to herpes-specific antiviral therapy. Past medical histories of mucocutaneous herpetic vesicular eruption, oral herpetic stomatitis, and herpetic blepharoconjunctivitis were all considered for the diagnosis. Previous or current clinical manifestations consisting of herpetic epithelial and/or stromal keratitis, characterized by dendritic or geographic ulceration, inflammatory stromal infiltration, stromal edema, and hypoesthesia were required for inclusion in the study. Corneal examination parameters performed on all patients included a refractive power analysis (OPD-Scan III, Nidek Co., LTD. Japan), a detailed comparative correlative slit lamp exam, including colored photographs and schematic drawings of the corneal lesions, as well as qualitative esthesiometry and fluorescein and lissamine green staining. Therapeutic response to conventional antiviral therapy was also considered necessary for the diagnosis.^[7,8][9]^

The exclusion criteria consisted of an unwillingness to participate in the study, any previous history of corneal pathology such as trauma, chemical burns, bacterial, fungal, adenoviral, or protozoan infection, and other forms of inflammatory or infectious external disease. Corneas with disciform keratitis, endotheliitis, anterior uveitis, or neurotrophic keratitis were excluded from the analysis. Additionally, cases with a doubtful diagnosis or with atypical HSK defined as corneas where dendritic or geographical ulcers, epithelial infiltration, and hypoesthesia were not evident were excluded from the analysis.

Before inclusion in the study, all patients read and signed an informed consent form previously approved by the Research and Ethics Committees of our institution according to the tenets of the Declaration of Helsinki.

Corneas included for analysis were divided according to the pathophysiologic classification into immunologic, or non-necrotizing, and infectious, or necrotizing, HSK.

Corneas were also categorized as inactive when corneal opacity and scarring due to herpetic keratitis was evident, and no signs or symptoms of inflammation or infection were present. Active disease was considered when patients experienced related symptoms, including red eye, blurred vision, foreign body sensations, photophobia, tearing, and pain. Additionally, signs of active inflammation, such as ciliary injection, corneal ulceration, active vascularization, inflammatory infiltration, stromal edema or melting, and keratic precipitates were observed under slit-lamp examination.

SD-OCT (RTVue-100Ⓡ, Optovue, Inc., Fremont, CA, USA) analysis was performed by the same technician (SIS) to each eye using a corneal module adaptor (CAM) L-lens (15 µm) and S-lens (10 µm), depending on whether the full extent or the details of corneal lesions, respectively, were analyzed as requested in a drawing scheme. While the wide-angle CAM L-lens provides a scan width of up to 6 mm and a transverse resolution of 15 μm, the high magnification CAM S-lens provides a scan width of up to 4 mm and a transverse resolution of 10 μm. Additionally, the corneal adaptor module software (version 5.5) automatically processes five consecutive sets of eight high-definition meridional scans, of which the three most consistent sets are used to provide a 6 mm scan diameter pachymetry map and the minimum corneal thickness point.^[20]^ Specific measurements of corneal epithelial and stromal thicknesses were also performed manually at the site of the lesions. Cursors were always placed perpendicular to the anterior corneal surface at the point of measurement. Four thickness measurements were considered for analysis: the central corneal thickness (CCT), minimal corneal thickness (MCT), corneal thickness at the site of the lesion (CTL), and corneal epithelial thickness over the stromal lesion (CETL).

Multiple high-resolution meridional scans were performed for each corneal lesion related to HSK according to a drawing scheme prepared from direct observations under the slit lamp. Representative scan images were selected for morphologic analysis by one of us (ARG) based on representative slit-lamp observations and corneal color photographs registered just before sending the patients for SD-OCT analysis. Corneal lesions consisting of inflammatory infiltrates and stromal edema from eyes with active keratitis and scars or leukomas from eyes with inactive disease were studied.

Stromal edema assessed by SD-OCT was defined as a hypodense area (grade I, < 33%) within the involved corneal stroma, accompanied by a ≥ 20% increase in thickness compared to the adjacent unaffected stroma. Stromal thinning was defined as a ≥ 15% thickness reduction compared to an immediate adjacent healthy area. Inflammatory infiltrate was described as a hyperdense lesion (grades II, 34–66%, and III, > 67%) within the involved corneal stroma, accompanied by a ≥ 10% increase in the thickness of an adjacent unaffected area.

The following measurement parameters were analyzed: localization (central = 5 mm zone, or paracentral/peripheral = 5–12 mm zone); percentage of surface extension (small ≤ 25%, medium = 25–45%, and large ≥ 45%); depth (superficial ≤ 150 µm, medial = 150–350 µm, and deep ≥ 350 µm); and density, which was analyzed using the color scale provided by the SD-OCT device. For this purpose, a classification system was created to quantify the density of each lesion by examining the warm tones (white, red, orange) that predominated in the image, which corresponded to the percentage of backscattering and reflective properties of the tissue analyzed (grade I, < 33%; grade II, 34–66%; and grade III, > 67%).

Statistical analysis was performed using the SPSS software version 23.0 (SPSS Inc. Chicago, IL. USA). A Shapiro-Wilk test was performed to explore the normality of variable distribution; observing that the pachymetric measurements, density, and depth values of the corneal lesions were not distributed normally, nonparametric tests (Mann–Whitney U-test) were used to compare the difference in medians. A *P*-value < 0.05 was considered as statistically significant.

### Ethical Consideration

Ethics Committee Approval No. CONBIOETICA19CEI00820130520)

Research Committee Approval No. 13C119039138

COFEPRIS Authorization No. 13CEI19039139

##  RESULTS

SD-OCT scanning was performed on 53 corneas of patients with unilateral HSK. These corneas belonged to 43 (81.1%) women and 10 (18.9%) men. The mean age at presentation was 41.0 years (range, 7 to 77 years).

Most eyes (83.1%) had immune or non-necrotizing HSK, and only nine (16.9%) eyes had the infectious or necrotizing form. Regarding the inflammatory status at the time of analysis, 24 (45.3%) eyes had active keratitis and 29 (54.7%) had inactive keratitis.

In the group of patients with active keratitis, 17 (70.8%) corneas belonged to women and 7 (29.1%) to men with a mean age of 42.2 years (range, 9–77 years). The most frequent symptoms found in these patients were red eye, tearing, and photophobia, and the most frequent signs were red eye, stromal edema, and hypoesthesia.

Meanwhile, in the inactive keratitis group, 25 (86.2%) corneas belonged to women and only 4 (13.7%) to men with a younger mean age of 39.9 years (range, 7–70 years). Apart from nonspecific symptoms of dry eye and discomfort, most of these patients were asymptomatic at the time of the analysis, and only those with corneal scarring along the visual axis or irregular astigmatism complained of blurred vision.

### SD-OCT Microstructural Analysis for Active Keratitis

Table 1 shows the frequency of the SD-OCT morphologic alterations from corneas with active keratitis. The most common findings were stromal edema and inflammatory infiltrates seen in 79.1% of eyes each, followed by epithelial thickening in 66.6% and stromal scarring in 62.5% of eyes. Of the seven corneas with active necrotizing HSK, five (71.4%) showed epithelial ulceration over the stromal infiltration. Half of the HSK lesions affected the visual axis, and the other 50% were located in the paracentral and peripheral cornea. Additionally, nearly half of the active corneal lesions were found at mid-stromal depth, had a medium density, and covered an area between 25 and 45% of the corneas (Table 2).

**Table 1:** Frequency of SD-OCT morphologic changes of herpetic stromal keratitis according to inflammatory status and type of keratitis.


**Corneal structure change (cross-sectional analysis)**	**Active HSK No. Eyes (%) (***n***** = 24)****	**Inactive HSK No. Eyes (%) (***n***** = 29)****	**** ***P*** **-value**	**Non-necrotizing keratitis No. Eyes (%) (***n***** = 44)****	**Necrotizing keratitis No. Eyes (%) (***n***** = 9)****	**** ***P*** **-value**
Epithelial remodeling	1 (4.1)	21 (72.4)	< 0.001*	19 (43.1)	3 (33.3)	0.02*
Epithelial thickening	16 (66.6)	1 (3.4)	< 0.001*	13 (29.5)	4 (44.4)	0.387
Epithelial damage (ulceration)	5 (20.8)	0 (0.0)	< 0.001*	0 (0.0)	5 (55.5)	< 0.001*
Stromal thinning (< 450 µm)	6 (25.0)	20 (68.9)	0.004*	20 (45.4)	7 (77.7)	0.080
Stromal edema (> 570 µm)	19 (79.1)	1 (3.4)	< 0.001*	15 (34.0)	4 (44.4)	0.652
Stromal inflammatory infiltration	19 (79.1)	2 (6.8)	< 0.001*	14 (31.8)	7 (77.7)	0.011*
Stromal scarring (> 35% surface)	15 (62.5)	26 (89.6)	0.042*	34 (77.2)	9 (100.0)	0.095
SD-OCT, spectral-domain optical coherence tomography; HSK, herpetic stromal keratitis ${}^{*}$ *P*-value < 0.05 was considered as statistically significant (Mann–Whitney U-test for two independent samples).						

**Table 2:** SD-OCT cross-sectional analysis of most representative corneal lesions (scar, leukoma, or infiltrate) seen in eyes with herpetic stromal keratitis.


**Lesion(s) parameter measured**	**Disease Activity**			**Type of Keratitis**		
	**Active HSK No. Eyes (%) (***n***** = 24)****	**Inactive HSK No. Eyes (%) (***n***** = 29)****	**** ***P-*** **value**	**Non necrotizing keratitis No. Eyes (%) (***n***** = 44 )****	**Necrotizing keratitis No. Eyes (%) (***n***** = 9 )****	**** ***P-*** **value**
**Localization:**			
- Central (visual axis)	12 (50.0)	15 (51.7)	0.901	20 (45.4)	7 (77.7)	0.08
- Paracentral/peripheral	12 (50.0)	14 (48.3)	0.901	24 (54.6)	2 (22.3)	0.08
**Density:**			
- Low (grade-I, < 33%)	7 (29.1)	10 (34.4)	0.464	17 (36.9)	0 (0.0)	0.040*
- Medium (grade-II, 34–66%)	11 (45.8)	13 (44.8)	0.942	21 (45.7)	3 (33.3)	0.956
- High (grade-III, > 67%)	6 (25.0)	6 (20.6)	0.777	8 (17.4)	6 (66.7)	0.010*
**Depth:**			
- Superficial (< 150 µm)	7 (29.2)	10 (34.5)	0.683	17 (38.6)	0 (0.0)	0.025*
- Medial (150–350 µm)	11 (45.8)	13 (44.8)	0.942	21 (47.7)	3 (33.3)	0.434
- Deep (> 350 µm)	6 (25.0)	6 (20.7)	0.712	6 (13.6)	6 (66.7)	0.001*
**Extension:**			
- Small (< 25% surface)	4 (16.6)	6 (20.6)	0.712	10 (22.7)	0 (0.0)	0.116
- Medium (25–45% surface)	13 (54.1)	21 (72.4)	0.172	30 (68.1)	4 (44.4)	0.18
- Large (> 45% surface)	7 (29.1)	2 (6.8)	0.033*	4 (9.0)	5 (55.5)	0.001*
SD-OCT, spectral-domain optical coherence tomography; HSK, herpetic stromal keratitis ${}^{*}$ *P*-value < 0.05 was considered as statistically significant (Mann–Whitney U test for two independent samples).			

**Table 3:** Mean pachymetry measurements by SD-OCT of corneas with herpetic stromal keratitis according to inflammatory status and type of keratitis.


	**Disease Activity**			**Type of Keratitis**		
**Measurement Parameter (µm)**	**Eyes with active keratitis (** ***n*** ** = 24)**	**Eyes with Inactive keratitis (** ***n*** ** = 29)**	**** ***P-*** **value**	**Non- necrotizing keratitis (** ***n*** ** = 44)**	**Necrotizing keratitis (** ***n*** ** = 9)**	**** ***P-*** **value**
**CCT**	561.7	482.3	< 0.001*	519.7	492.8	O.405
**MCT**	483.5	436.4	0.007*	468.9	396.3	0.002*
**CTL**	644.7	439.9	< 0.001*	528.2	473.5	0.711
**CETL**	68.7	78.4	0.048*	72.2	47.1	0.043*
**Stromal thinning (irregular astigmastism** ${}^{†}$ **) No eyes (%)**	5 (20.8%)	11 (37.9%)${}^{†}$	0.181	6 (13.6%)	5 (62.5%)${}^{†}$	0.001*
SD-OCT, spectral-domain optical coherence tomography; CCT, central corneal thickness; MCT, minimum corneal thickness; CTL, corneal thickness at lesion site; CETL, corneal epithelium thickness at lesion site; ${}^{†}$confirmed by topography ${}^{*}$ *P*-value < 0.05 was considered as statistically significant (Mann–Whitney U test for two independent samples).				

**Figure 1:**
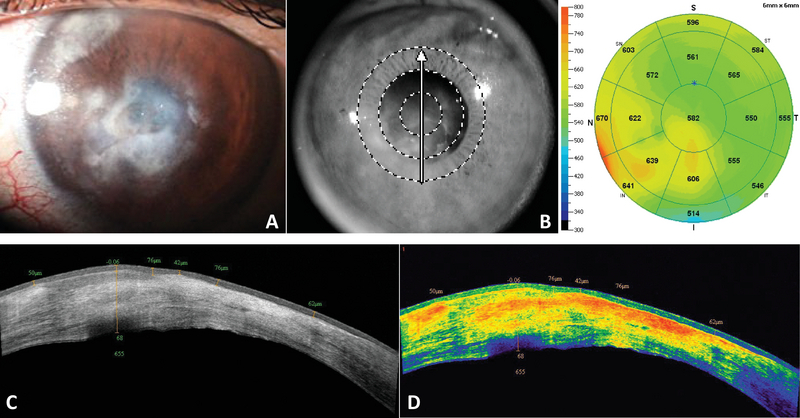
(A) Slit-lamp photograph of a cornea with active necrotizing HSK showing stromal inflammatory infiltration and edema. (B) SD-OCT pachymetry map showing a thickened cornea (max = 670 µm) corresponding to the area with stromal edema. (C) A cross-sectional view of the central lesion showing areas of epithelial thickening (max = 76 µm) over inflammatory infiltrates, corresponding to areas of hyperreflectivity, and hypodense areas, corresponding to stromal edema. (D) Color scale of the same lesion showing the inflammatory infiltrates and scarring in warm (orange to yellow) tones.

The corneal shape was distorted within the affected zones, showing a hypodense and thickened stroma due to edema. There was also a higher reflectivity at the site of stromal inflammatory infiltrates compared to unaffected areas (Figure 1).

**Figure 2:**
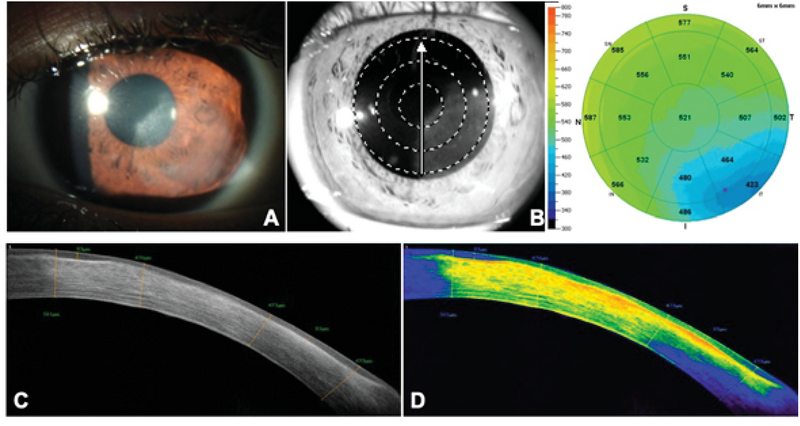
(A) Slit-lamp photograph of a cornea with inactive nonnecrotizing HSK, showing a diffuse leukoma with no stromal edema. (B) SD-OCT pachymetry showing a thinner cornea (min = 423 µm) in the area of the lesion. (C) A cross-sectional view of the leukoma showing areas of epithelial remodeling and thickening compensation (max = 83 µm) under areas of scarring and stromal compaction (min = 470 µm). (D) Color scale of the lesion showing higher density areas corresponding to fibrosis and scarring on the anterior and medial stroma, fading to yellow and green tones as the lesion becomes less dense.

**Figure 3:**
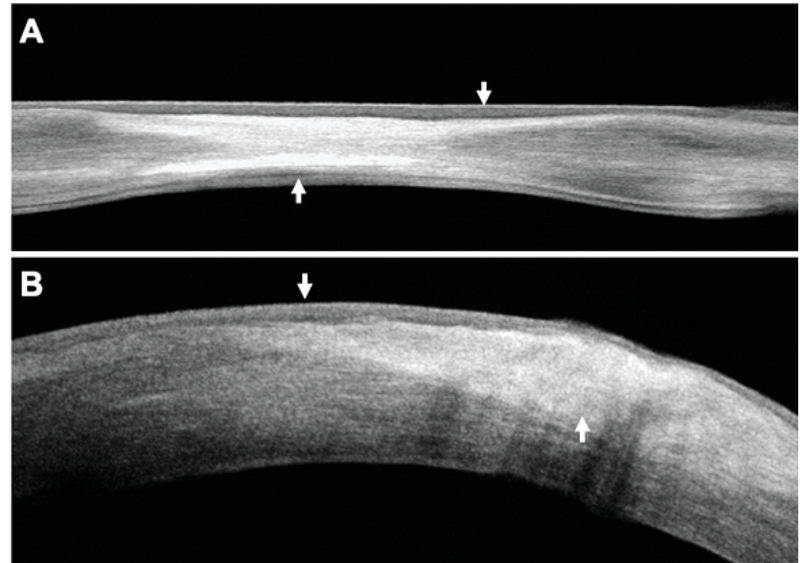
(A) A cross-sectional image of a cornea with inactive HSK showing epithelial remodeling (top arrow) over a zone of stromal fibrosis and compaction (bottom arrow) with the MCT = 441 µm. (B) A cross-sectional image of a cornea with active HSK showing marked stromal edema (CTL = 641 µm) and an active inflammatory infiltrate (bottom arrow) underlying an area of epithelial edema.

### SD-OCT Microstructural Analysis for Inactive Keratitis

The most common SD-OCT morphologic changes seen in corneas with inactive keratitis consisted of stromal scarring (89.6%), epithelial remodeling (72.4%), and stromal thinning (68.9%) (Table 1).

Similar to the eyes with active HSK, nearly half of the lesions were located in the visual axis, and the other 48.3% were located in the paracentral and peripheral cornea. Additionally, 44.8% of the lesions extended to the mid-stromal depth and had a medium density. Finally, most of the inactive lesions (72.4%) covered an area between 25 and 45% of the ocular surface (Table 2). In general, the SD-OCT analysis of inactive HSK showed thinner corneas with stromal compaction due to fibrosis and scarring. The overlying epithelium was thicker in the affected areas, showing remodeling and thickness compensation. Additionally, there was a higher reflectivity, particularly in areas of dense leukomas (Figure 2).

### SD-OCT Microstructural Differences Between Non-necrotizing and Necrotizing HSK

There was a clear difference in the morphologic appearance between non-necrotizing and necrotizing keratitis. All corneas with necrotizing HSK showed severe stromal scarring, and the vast majority also had significant stromal inflammatory infiltration and thinning (Table 1). On the other hand, the non-necrotizing corneas showed significantly less inflammatory infiltration (*P* = 0.011).

Following the SD-OCT cross-sectional analysis, the majority of necrotizing HSK lesions (77.7%) were located centrally, affecting the visual axis, and showed a higher density compared to non-necrotizing lesions (*P* = 0.01) (Table 2). Additionally, 66.7% of the lesions analyzed were located deep into the corneal stroma (*P* = 0.001), and many of them covered a significant area of the corneal surface (*P* = 0.001). By contrast, most non-necrotizing lesions were in the low- to medium-density range, extended between the superficial to medium stromal depths, and affected a smaller area of the cornea than in necrotizing keratitis (Table 2).

### Pachymetry Comparative Analysis 

The comparative analysis of corneal thickness between the active and inactive keratitis groups showed significant differences in the four measured parameters: CCT, MCT, CTL, and CETL (Table 3). Eyes with inactive HSK had significant stromal thinning compared to those with active disease (*P* ≤ 0.007). However, the corneal epithelial thickness at the site of the lesion was significantly increased in the inactive cases (*P =* 0.048). On the other hand, the values of MCT (*P =* 0.002) and CETL (*P* = 0.043) were significantly lower in corneas with necrotizing keratitis than in non-necrotizing keratitis. Finally, almost one-third of the eyes showed irregular astigmatism and severe stromal thinning with a major predominance in the necrotizing group (*P* < 0.001).

##  DISCUSSION

Clinically, the corneal pathologic characteristics seen in HSK depend on direct slit-lamp observation of inflammatory stromal infiltration, edema, neovascularization, scarring, and thinning with a history of herpes infection and previously known herpetic epithelial ulceration and corneal hypoesthesia.^[15,23]^

SD-OCT allows an *in vivo*, accurate, noncontact, and fast examination of the entire corneal microstructure in different inflammatory pathologies, including HSK.^[24,25,26]^ To the best of our knowledge, no systematic microstructural analyses have been performed of the corneal pathologic changes seen in HSK using SD-OCT. In the present study, we described these changes in different stages of disease activity and severity. Spectral-domain tomography permits a detailed analysis of the entire cornea in cross-sections of variable orientations as well as en face fragmentation analysis.^[24]^ Most corneas analyzed in the inactive stage of the disease showed epithelial remodeling, consisting of thickening over the scarred stroma that was characterized by fibrosis, thinning, and compaction (Figure 2).

In contrast, more than two-thirds of the corneas from patients with active keratitis showed significant stromal edema characterized by increased CCT and CTL (Table 3), as well as a hypodense and thickened stromal appearance (Figure 1). In general, patients with the inactive stage of HSK did not show corneal stromal edema; their CCT measurements were in the low to normal range (356–537 µm), and the mean CTL was even lower (439.9 µm), corresponding to areas of stromal scarring and thinning.

Corneas from the active keratitis group showed a thicker epithelium than average at the site of the lesion (mean CETL = 68.7 µm) and a significantly increased corneal thickness (mean CTL = 644.7 µm), reflecting the presence of stromal inflammatory infiltration and edema.

Of note was the occurrence of severe corneal thinning in a total of 16 (30.1%) eyes with HSK, with a higher proportion in corneas with necrotizing disease (Table 3). Necrotizing HSK, the most severe form of herpetic keratitis, is characterized by significant leukocyte stromal infiltration and tissue necrosis with consequent thinning of the cornea, which explains the higher percentage of severe corneal thinning and irregular astigmatism seen in these corneas.^[7,27]^

SD-OCT measures the intensity of a backscattered optical signal, which represents the reflectivity of the tissue.^[28]^ Since reflectivity varies among different tissues, we can differentiate them by measuring their reflectivities in transverse scans that can be displayed as false-color or grayscale cross-sectional images.^[29,30]^ The intensity of the backscattered optical signal is represented on a logarithmic scale with varying degrees of brightness. White corresponds to the highest reflection, while dark gray and black correspond to weaker back reflection.^[30]^ SD-OCT software allows toggling between gray- and color scales for each image created. The color scale allows easier detection of subtle variations in the OCT signal to define reflectivity levels. Conversely, the grayscale allows the viewer to visualize contrast more easily.^[31]^ We used the color scale to measure the density of inflammatory infiltrates in eyes with active keratitis and leukomas or scarring in eyes with inactive HSK. In both active and inactive disease, approximately two-thirds of the eyes showed medium to high density lesions; however, corneas with necrotizing stromal keratitis had a significantly higher percentage of high-density lesions (Table 2).

We found a particular limitation in differentiating between stromal edema and inflammatory infiltration in corneas with active keratitis, where both lesions coexisted within the same stromal area. However, differences in reflectivity, where stromal edema appears as a hypodense area of increased stromal thickness and low reflectivity and stromal infiltrates as areas of increased stromal thickness but with higher reflectivity (yellow-to-orange color), help to facilitate their differentiation.

Since there are no previous specific reports on SD-OCT findings in this pathologic condition, we find the present study of value to improve our understanding of the *in vivo* pathologic changes occurring at different stages and severity of HSK. As in other corneal pathologies, we consistently found epithelial remodeling, consisting of zonal thickening at sites of fibrosis and scarring in inactive disease (Figure 3A). Epithelial remodeling has also been observed as a way of anterior curvature compensation in advanced keratoconus under areas of significant stromal thinning and ectasia.^[32]^ In contrast, epithelial thickening was present in most eyes during active inflammation (Figure 3B).

In a clinical setting, corneal SD-OCT could be useful to analyze scarring extension and leukoma depth, hence facilitating surgical decisions regarding partial or total corneal transplantation. Additionally, it may be useful for the detection and grading of stromal edema during recurrent inflammation under challenging situations. During active HSK, leucocyte infiltration induces stromal edema characterized by diffuse haziness, giving a “ground glass” appearance in the area surrounding the inflammatory infiltrate. Subtle stromal edema may hide in the infiltrate or under a dense leukoma. In such circumstances, corneal SD-OCT represents a useful aid for its detection. The potential limitations of the present study include distortions that occur in the SD-OCT scans as a result of the refractive indexes of the tissues. The acquisition of SD-OCT scans was performed along meridional planes to minimize this effect, and all images were de-warped by the SD-OCT system's corneal adaptor module software.^[31]^

In conclusion, *in vivo* SD-OCT pathologic analysis of corneas with different disease activity and types of inflammation has permitted a better understanding of the pathologic mechanisms adopted by the affected corneas. Additionally, it allowed the evaluation of the grade and extent of tissue damage and repair seen in HSK. Future studies of HSK imaging analysis with SD-OCT should be carried out to monitor disease progression or therapeutic response, including OCT angiography of the cornea to analyze blood perfusion and neovascularization responses. Additionally, the commercial development of ultra-high-definition OCTs for corneal and anterior segment imaging analysis surely would yield more detailed *in vivo* ultrastructure pathologic changes occurring in this sight-threatening disease.

##  Acknowledgements

The authors are thankful to Susana Imperial-Sauceda for her technical support in performing all corneal SD-OCT tests.

##  Financial Support and Sponsorship

This study has been partially funded by the Immuneye Foundation.

##  Conflicts of Interest

There are no conflicts of interest.

## References

[1] Liesegang TJ. Herpes simplex virus epidemiology and ocular importance. *Cornea *2001;20:1–13. 10.1097/00003226-200101000-00001 11188989

[2] Labetoulle M, Auquier P, Conrad H, Crochard A, Daniloski M, Bouée S, et al. Incidence of herpes simplex virus keratitis in France. *Ophthalmology* 2005;112:888–895. 10.1016/j.ophtha.2004.11.052 15878072

[3] Wilhelmus K, Beck RW, Moke PS, Dawson CR, Barron BA, Jones DB, et al. Acyclovir for the prevention of recurrent herpes simplex virus eye disease. *N Engl J Med* 1998; 339:300–306. 10.1056/NEJM199807303390503 9696640

[4] Wilhelmus K, Dawson C, Barron B, Bacchetti P, Gee L, Jones DB, et al. Risk factors for herpes simplex virus epithelial keratitis recurring during treatment of stromal keratitis or iridocyclitis. Herpetic Eye Disease Study Group. *Br J Ophthalmol* 1996;80:969–972. 10.1136/bjo.80.11.969 PMC505673 8976723

[5] Farooq AV, Shukla D. Herpes simplex epithelial and stromal keratitis: an epidemiologic update. *Surv Ophthalmol* 2012;57:448–462. 10.1016/j.survophthal.2012.01.005 PMC3652623 22542912

[6] Barron BA, Gee L, Hauck WW, Kurinij N, Dawson CR, Jones DB, et al. Herpetic eye disease study: a controlled trial of oral acyclovir for herpes simplex stromal keratitis. *Ophthalmology* 1994;101:1871–1882. 10.1016/s0161-6420(13)31155-5 7997323

[7] Holland EJ, Schwartz GS. Classification of herpes simplex virus keratitis. *Cornea* 1999;18:144–154. 10.1097/00003226-199903000-00002 10090359

[8] Wilhelmus KR. Diagnosis and management of herpes simplex stromal keratitis. *Cornea* 1987;6:286–291. 10.1097/00003226-198706040-00011 3319411

[9] Hendricks RL, Tumpey TM. Contribution of virus and immune factors to herpes simplex virus type I-induced corneal pathology. *Invest Ophthalmol Vis Sci* 1990;31:1929–1939. 2170289

[10] Streilein JW, Dana MR, Ksander BR. Immunity causing blindness: five different paths to herpes stromal keratitis. *Immunol Today* 1997;18:443–449. 10.1016/s0167-5699(97)01114-6 9293161

[11] Remeijer L, Osterhaus A, Verjans G. Human herpes simplex virus keratitis: the pathogenesis revisited. *Ocul Immunol Inflamm* 2004;12:255–285. 10.1080/092739490500363 15621867

[12] Brik D, Dunkel E, Pavan-Langston D. Herpetic keratitis: persistence of viral particles despite topical and systemic antiviral therapy. *Arch Ophthalmol* 1993;111:522–527. 10.1001/archopht.1993.01090040114043 8385924

[13] Holbach LM, Font RL, Baehr W, Pittler SJ. HSV antigens and HSV DNA in avascular and vascularized lesions of human herpes simplex keratitis*. Curr Eye Res* 2009;10:63–68. 10.3109/02713689109020359 1650674

[14] Holbach LM, Font RL, Naumann GO. Herpes simplex stromal and endothelial keratitis: granulomatous cell reactions at the level of Descemet´s membrane, the stroma, and Bowman´s layer. *Ophthalmology* 1990;97:722–728. 10.1016/s0161-6420(90)32524-1 2165231

[15] Wilhelmus KR, Mitchell BM, Dawson CR, Jones DB, Barron BA, Kaufman HE, et al. Slit-lamp biomicroscopy and photographic image analysis of herpes simplex virus stromal keratitis. *Arch Ophthalmol* 2009;127:161–166. 10.1001/archophthalmol.2008.577 19204233

[16] Hamrah P, Sahin A, Dastjerdi MH, Shahatit BM, Bayhan HA, Dana R, et al. Cellular changes of the corneal epithelium and stroma in herpes simplex keratitis. An in vivo confocal microscopy study. *Ophthalmology* 2012;119:1791–1797. 10.1016/j.ophtha.2012.03.005 PMC3426622 22608476

[17] Hillenaar T, van Cleynenbreugel H, Verjans GM. Monitoring the inflammatory process in herpetic stromal keratitis: the role of in vivo confocal microscopy. *Ophthalmology* 2012;119:1102–1110. 10.1016/j.ophtha.2011.12.002 22361312

[18] Khurana RN, Li Y, Tang M, Lai MM, Huang D. High speed optical coherence tomography of corneal opacities. *Ophthalmology *2007;114:1278–1285. 10.1016/j.ophtha.2006.10.033 17307254

[19] de Boer JF, Cense B, Park BH, Pierce MC, Tearney GJ, Bouma BE. Improved signal-to-noise ratio in spectral-domain compared with time-domain optical coherence tomography. *Opt Lett* 2003;28:2067–2069. 10.1364/ol.28.002067 14587817

[20] Binder P, Trokel S, Salaroli C, Li Y, Yiu S, Ramos S, et al. Corneal pathologies and surgeries. In: Huan D, editor. RTVue fourier-domain optical coherence tomography primer series, vol. 2, 2nd edn. Fremont, CA, USA: Optovue Inc.; 2009:46–53.

[21] Huang D, Swanson EA, Lin CP, Schuman JS, Stinson WG, Chang W, et al. Optical coherence tomography. *Science* 1991;254:1178–1181. 10.1126/science.1957169 PMC4638169 1957169

[22] Li Y, Shekhar R, Huang D. Corneal pachymetry mapping with high-speed optical coherence tomography. *Ophthalmology* 2006;113:792–799. 10.1016/j.ophtha.2006.01.048 PMC1474520 16650675

[23] Pepose JS, Keadle TL, Morrison LA. Ocular herpes simplex: changing epidemiology, emerging disease patterns, and the potential of vaccine prevention and therapy. *Am J Ophthalmol* 2006;141:547–557. 10.1016/j.ajo.2005.10.008 16490506

[24] Wirbelauer C. OCT of corneal opacities. *Ophthalmology* 2008;115:589–590. 10.1016/j.ophtha.2007.08.026 18319112

[25] Sandali O, El Sanharawi M, Temstet C, Hamiche T, Galan A, Ghouali W, et al. Fourier-domain optical coherence tomography imaging in keratoconus: a corneal structural classification. *Ophthalmology* 2013;120:2403–2412. 10.1016/j.ophtha.2013.05.027 23932599

[26] Dhaini AR, Fattah MA, El-Oud SM, Awwad ST. Automated detection and classification of corneal haze using optical coherence tomography in patients with keratoconus after cross-linking. *Cornea *2018;37:863–869. 10.1097/ICO.0000000000001570 29538101

[27] Heiligenhaus A, Bauer D, Meller D, Steuhl KP, Tseng SC. Improvement of HSV-1 necrotizing keratitis with amniotic membrane transplantation. *Invest Ophthalmol Vis Sci *2001;42:1969–1974. 11481259

[28] Mahdian, Mina, "Tissue Characterization Using Optical Coherence Tomography" (2015).Master's Theses. 793. https://opencommons.uconn.edu/gs_theses/793

[29] Knuettel AR, Bonev S, Knaak W. New method for evaluation of in vivo scattering and refractive index properties obtained with optical coherence tomography. *J Biomed Optometry* 2004;9:265–273. 10.1117/1.1647544 15065890

[30] Kholodnykh AI, Petrova IY, Larin KV, Motamedi M, Esenaliev RO. Precision of measurement of tissue optical properties with optical coherence tomography. *Appl Optomet* 2003;42:3027–3037. 10.1364/ao.42.003027 12790454

[31] Wojtkowski M, Bajraszewski T, Targowski P, Kowalczyk A. Real-time in vivo imaging by high-speed spectral optical coherence tomography. *Opt Lett* 2003;28:1745–1747. 10.1364/ol.28.001745 14514087

[32] Sandali O, El Sanharawi, Temstet C, Hamiche T, Galan A, Ghouali W, et al. Fourier-domain optical coherence tomography imaging in keratoconus. A corneal structural classification. *Ophthalmology *2013;120:2403–2412. 10.1016/j.ophtha.2013.05.027 23932599

